# Non-alcoholic fatty liver and fibrosis is associated with cardiovascular structure and function in young adults

**DOI:** 10.1097/HC9.0000000000000087

**Published:** 2023-03-30

**Authors:** Rosalind Tang, Kushala W. M. Abeysekera, Laura D. Howe, Alun D. Hughes, Abigail Fraser

**Affiliations:** 1 Guy’s & St Thomas’ NHS Foundation Trust, London, UK; 2 Bristol Medical School, Faculty of Health Sciences, University of Bristol, Bristol, UK; 3 Keenan Research Centre for Biomedical Science, St Michael’s Hospital, Toronto, Ontario, Canada; 4 Population Health Sciences, Bristol Medical School, University of Bristol, Bristol, UK; 5 Department of Liver Medicine, University Hospitals Bristol and Weston NHS Foundation Trust, Bristol, UK; 6 MRC Integrative Epidemiology Unit, University of Bristol, Bristol, UK; 7 Population Science & Experimental Medicine, Institute of Cardiovascular Science, University College, London, London, UK

## Abstract

Non-alcoholic fatty liver disease shares many risk factors with other metabolic disorders. We sought to establish whether non-alcoholic fatty liver disease may be associated with cardiovascular health independently of other known risk factors.

**Methods:**

In this prospective, population-based cohort of young adults, controlled attenuation parameter-defined liver steatosis, transient elastography-defined liver fibrosis, echocardiography, carotid ultrasonography, and pulse wave analysis were assessed at age 24 years. We examined associations between liver and cardiovascular measures, with and without accounting for demographics, body mass index, alcohol, smoking, blood pressure, lipidemia, glycemia, and inflammation.

**Results:**

We included 2047 participants (mean age 24.4 y; 36.2% female): 212 (10.4%) had steatosis, whereas 38 (1.9%) had fibrosis. Steatosis was associated with cardiovascular measures after adjusting for demographics, but with more comprehensive adjustment, steatosis only remained associated with stroke index [β (95% CI) of −1.85 (−3.29, −0.41) mL/m^2^] and heart rate [2.17 (0.58, 3.75) beats/min]. Fibrosis was associated with several measures of cardiovascular structure and function after full adjustment for risk factors, including left ventricular mass index [2.46 (0.56, 4.37) g/m^2.7^], E/A ratio [0.32 (0.13, 0.50)], tricuspid annular plane systolic excursion [0.14 (0.01, 0.26) cm], carotid intima-media thickness [0.024 (0.008, 0.040) mm], pulse wave velocity [0.40 (0.06, 0.75) m/s], cardiac index [−0.23 (−0.41, −0.06) L/min⋅m^2^], and heart rate [−7.23 (−10.16, −4.29) beats/min].

**Conclusions:**

Steatosis was not associated with measures of cardiovascular structure and function nor with subclinical atherosclerosis after adjusting for known cardiovascular risk factors. Fibrosis, however, was associated with several cardiovascular measures, including indicators of subclinical atherosclerosis, even after full adjustment. Further follow-up will help determine whether cardiovascular health worsens later with steatosis alone.

## INTRODUCTION

Non-alcoholic fatty liver disease (NAFLD), diabetes, and cardiovascular disease have common risk factors, including age, sex, body mass index (BMI)/waist circumference, smoking, alcohol, blood pressure, lipids, glucose, insulin, and inflammation.^1^–^4^ Numerous studies have reported associations of NAFLD with cardiovascular disease and subclinical atherosclerosis in older adults, but few adjusted comprehensively for these shared risk factors.^2^,^4^,^5^ Of those studies that did account for confounders, some^2^,^4^ but not all^5^ suggest that NAFLD may be an independent risk factor for developing cardiovascular disease beyond their shared cardiometabolic risk factors.

There is also some evidence of associations between NAFLD and cardiac structure and function, even after accounting for age, ethnicity, sex, education, income, alcohol, smoking, physical activity, blood pressure, lipids, diabetes, and kidney function.^6^–^11^

The prevalence of NAFLD is increasing in young adults,^12^–^16^ but whether it is accompanied by atherosclerosis and/or impaired cardiac structure and function, once common risk factors are accounted for, remains unclear.^12^,^17^–^22^ The few pediatric studies of NAFLD and atherosclerosis have been reliant on low-sensitivity modalities of assessing NAFLD (ie, ultrasonography and/or laboratory test-based composite scores).^12^ Finally, few studies have been able to investigate NAFL steatosis and non-alcoholic steatohepatitis (NASH) fibrosis separately.^13^

We therefore investigated the associations of hepatic steatosis and fibrosis with cardiovascular measures obtained using echocardiography, carotid ultrasonography, and pulse wave analysis in a general population cohort of young adults.

## METHODS

### Study population

The Avon Longitudinal Study of Parents and Children (ALSPAC) is a prospective, population-based cohort study in the south west of the UK, which recruited 14,541 pregnant women with expected delivery dates between April 1, 1991, to December 31, 1992.^23^,^24^ The study included 13,988 children alive at 1 year and added 913 children following further recruitment after the age of 7 years.^23^,^24^ ALSPAC invited all 10,018 still-actively engaged participants to attend the 24-year assessment, and 4021 (40.1%) attended between June 5, 2015, and October 31, 2017.^20^–^22^ Data collection in ALSPAC is ongoing through clinic visits, questionnaires, and data linkages to external organizations. Ethical approval for the study was obtained from the ALSPAC Ethics and Law Committee and the Local Research Ethics Committees. Informed consent was obtained in writing from participants following the recommendations of the ALSPAC Ethics and Law Committee (Bristol, UK). All work was conducted in accordance with both the Declarations of Helsinki and Istanbul, and all data were collected and managed using REDCap electronic data capture tools hosted at the University of Bristol (Bristol, UK). The consent for biological samples has been collected in accordance with the Human Tissue Act (2004). Please note that the ALSPAC website contains the details of all available data through a fully searchable data dictionary and variable search tool (http://www.bristol.ac.uk/alspac/researchers/our-data/).

**FIGURE 1:**
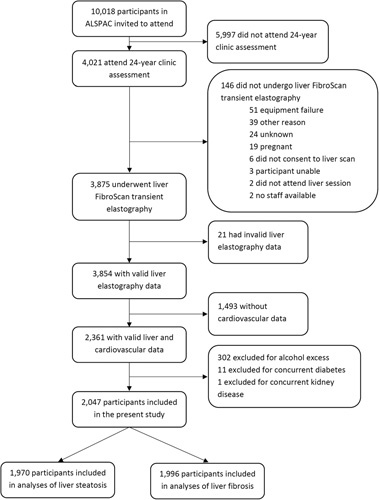
Flowchart of study participants. ALSPAC, Avon Longitudinal Study of Parents and Children.

In the present study, we included any ALSPAC participants who had liver transient elastography data at the 24-year clinic visit and who had at least 1 of 3 cardiovascular assessments: echocardiography, carotid ultrasonography, and/or pulse wave analysis. No participants reported viral hepatitis infection nor used medications for viral hepatitis. We excluded participants with the *Diagnostic and Statistical Manual of Mental Disorders 5th Edition* 25 criteria for alcohol use disorder, daily consumption of 6 or more units of alcohol even in the absence of alcohol use disorder symptoms, concurrent diabetes, and/or concurrent renal disease. Pregnant participants were excluded from undergoing ultrasonography (liver, cardiac, and carotid) in ALSPAC and therefore could not be included in the present study (Figure 1).

### Transient elastography

Fasting (≥6 h) transient elastography was performed at the 24-year visit using FibroScan 502 Touch (Echosens, Paris, France), as described by Abeysekera et al.^15^ ALSPAC intended to exclude participants with pacemakers or ascites from elastography, but no participants presented with either exclusion criterion. Liver data include controlled attenuation parameter (dB/m) as a measure of steatosis^26^ and liver stiffness as a measure of fibrosis (kPa).^27^ Controlled attenuation parameter measurements outside the 100–400 dB/m range were discarded, and fibrosis measurements were discarded if the interquartile-range-to-median ratio was ≥30%. The values were also discarded if <10 readings could be attained. These continuous variables were dichotomized for analysis (≤275 dB/m for no steatosis >275 dB/m for steatosis^26^ and <7.9 kPa for no fibrosis, and ≥7.9 for fibrosis^27^).

### Echocardiography

Echocardiography was measured by 2 sonographers using a Philips EPIQ 7G ultrasound and X5-1 transducer (Koninklijke Philips NV, Amsterdam, the Netherlands) in a different ALSPAC subset, although there was a large proportion of overlap with those with liver data. M-mode was used to calculate tricuspid annual plane systolic excursion (TAPSE). Two-dimensional images from the parasternal long axis and short axis and apical 2, 3, 4, and 5 chamber views were used to measure end-diastolic and end-systolic left ventricular (LV) dimensions (intraventricular septum, posterior wall thickness, LV inner dimension, and LV outflow tract area). Pulsed Doppler was used to assess transmitral flow peak early diastolic velocity (E wave) and peak late diastolic velocity (A wave), deceleration time, and aortic velocity time integral. Tissue Doppler imaging from the apical 4 and 5 chamber views assessed longitudinal tissue velocity at the mitral valve, including tricuspid lateral annular systolic velocity (s’) and early diastolic velocity (e’) for myocardial relaxation. Five cardiac cycles were recorded unless the participant was in atrial fibrillation, in which 10 cardiac cycles were recorded. Philips Q-station software (Koninklijke Philips NV, Amsterdam, the Netherlands) was used for M-mode, 2-dimensional, and tissue Doppler imaging measurements, whereas TOMTEC software (TOMTEC, Unterschleissheim, Germany) was used for the 3dimensional measurements of the left ventricle from the apical view.

We also calculated stroke volume (SV), cardiac output (CO), LV mass, and relative wall thickness (RWT) at end-diastole using the following equations:


SV=LVOTarea×LVOTVTI

CO=heartrate×SV

LVmass=0.8×(1.04×[(IVSd+LVIDd+PWTd)3−LVIDd3])+0.6

RWTd=(2×PWTd)/LVIDd

For all analyses, we normalized LV mass to height^2.7^, left atrium anteroposterior diameter to height, and LV end-diastolic volume, stroke volume, and cardiac output to body surface area (BSA; using the Dubois equation).

We examined echocardiography indicators of LV structure [end-diastolic volume index, RWT, LV mass index (LVMI)], LV systolic function (ejection fraction, s’ velocity), LV diastolic function (E/e’ ratio for left-sided filling pressures, e’ velocity for myocardial relaxation, left atrium anteroposterior diameter index, E/A ratio, transmitral E deceleration time), right ventricular systolic function (TAPSE), and hemodynamic measures (stroke index, cardiac index) as dependent variables.

### Carotid ultrasonography

Both right and left common carotid arteries were imaged 1 cm proximal to the carotid bifurcation using a CardioHealth Station ultrasound and a 13.5 MHz linear transducer (Panasonic Healthcare Group, Tokyo, Japan) with patients positioned supine. End-diastolic carotid intima-media thickness (cIMT) was measured on the posterior walls of both right and left common carotid arteries over 3 consecutive cardiac cycles. Any cIMT measurement >1.0 mm was reviewed for validity. Carotid-femoral pulse wave velocity (PWV) was measured using a Vicorder (Skidmore Medical Ltd, Bristol, UK) after participants rested semiprone with shoulders and head raised ~30 degrees for 5 minutes. PWV measurements were repeated until 3 values were within 0.5 m/s of each other.

### Pulse wave analysis and blood pressures

Central systolic and diastolic blood pressures were measured at the right radial artery using a SphygmoCor (AtCor Medical, Naperville, IL) calibrated to peripheral blood pressure. Peripheral blood pressure and heart rate were measured using an Omron M6 (OMRON Healthcare Ltd, Milton Keynes, UK). Systemic vascular resistance (SVR) was calculated as:


Systemicvascularresistance=meanarterialpressure/cardiacoutput

We also indexed SVR and total arterial compliance to BSA for all analyses.

### Covariates

Participants’ age, sex, ethnicity, and parents’ highest occupational social class at the time of birth were reported by parents/carers. Not in education, employment, or training (NEET) status; alcohol intake; and smoking behavior were self-reported through a questionnaire at the 24-year visit. Height, weight, fasting blood samples, and liver function tests were also assessed at this visit. In all analyses, triglycerides, insulin, glucose, and C-reactive protein (CRP) were log-normalized. Physical activity was measured using ActiGraph GT3X+ accelerometers (ActiGraph Corp, Pensacola, USA) over a consecutive 4-day period and included in sensitivity analyses as a continuous variable of counts per minute.

### Multiple imputation

Missing data ranged from zero to 27.1% across variables included in our analyses (Supplemental Table S1, http://links.lww.com/HC9/A223). To account for missing data in dependent variables and covariates to minimize the selection bias and increase power, we used multiple imputation by chained equations^28^ in Stata/MP 15 (StataCorp LLC, College Station) to impute 20 data sets. Supplemental Table S2 (http://links.lww.com/HC9/A223) reports the variables and regression models in our imputation. We included variables used in our analyses (independent variables, dependent variables, and covariates), as well as 12 auxiliary variables that are associated with the variables in our analyses or predictors of missingness.^28^ We selected auxiliary variables, which were known to be highly correlated with the incomplete variables in our analyses and those which are observed where our analysis variables are missing (alanine transaminase, gamma-glutamyl transferase, peripheral systolic and diastolic blood pressures, and blood tests from the 17-year clinic assessment: fasting triglycerides, fasting total cholesterol, fasting glucose, fasting insulin, alanine transaminase, gamma-glutamyl transferase, and CRP), as this reduces bias and improves precision when compared with complete case analysis.^28^ The distributions of data in the observed and imputed data sets were similar (Supplemental Table S1, http://links.lww.com/HC9/A223).

### Statistical analysis

We used linear regression to examine associations between steatosis and fibrosis, with measures from echocardiography, carotid ultrasonography, and pulse wave analysis. We then incrementally adjusted for demographics (age, sex, ethnicity, parents’ highest social class, and NEET status; results in Supplemental Table S3, http://links.lww.com/HC9/A223); demographics and confounders (BMI, alcohol, and smoking) in model 1; and the covariates in model 1, as well as additional confounders and potential mediators in our fully adjusted model 2 [mean arterial pressure (MAP), lipids, glucose, insulin, and CRP]. MAP was excluded as a covariate in the analyses of MAP and SVRI. Physical activity data were only available in 681/2047 (32.8%) participants. We therefore performed sensitivity analyses in this subset, adjusting for all covariates in model 3 and then adding in physical activity.

We report all estimates from multiple linear regression as mean differences (β) in the dependent variable with 95% CI. All statistical analyses were performed using Stata/MP 15 (StataCorp LLC, College Station).

## RESULTS

### Participants

A study flowchart is presented in Figure 1, and a distribution of participant characteristics is reported in Table 1. We included 2047 young adults (36.2% female) with a mean (SE) age of 24.4 (0.01) years. Of the 1970 with valid steatosis measures, 212 (10.4%) had steatosis (CAP>275 dB/m), whereas 38 (1.9%) of 1996 with valid fibrosis measures had fibrosis (liver stiffness ≥7.9 kPa).

**TABLE 1:** Characteristics of study participants (N=2047)

Characteristic	Mean (SE) or N (%)
Age, y	24.36 (0.01)
Female sex	740 (36.2)
Non-White ethnicity	57 (3)
Highest parental social class	
I	391 (19)
II	970 (48)
III (non-manual)	504 (24)
III (manual)	125 (6)
IV	42 (2)
V	15 (1)
Not in education, employment or training	158 (8)
Body mass index, kg/m^2^	24.7 (0.1)
Alcohol units per day of drinking	
0–2	599 (29)
3–6	1063 (52)
7 or more	385 (19)
Smoking status	
Never smoker	858 (42)
Ex-smoker	737 (36)
Current smoker	452 (22)
Fasting triglycerides, mmol/L	0.95 (0.01)
Fasting total cholesterol, mmol/L	4.39 (0.02)
Fasting HDL cholesterol, mmol/L	1.54 (0.01)
Fasting glucose, mmol/L	5.3 (0.01)
Fasting insulin, mIU/L	10.0 (0.2)
C-reactive protein, mg/L	2.2 (0.2)
Exposures	
Controlled attenuation parameter (CAP), dB/m	208.1 (1.2)
Steatosis	
CAP ≤275 dB/m	1757 (86)
CAP >275 dB/m	212 (10)
Missing	77 (4)
Liver stiffness, kPa	4.75 (0.03)
Fibrosis	
Liver stiffness <7.9 kPa	1958 (96)
Liver stiffness ≥7.9 kPa	38 (2)
Missing	51 (2)
Cardiovascular outcomes	
Left ventricular structure	
End-diastolic volume index, mL/m^2^	55.7 (0.3)
Relative wall thickness at end diastole	0.366 (0.002)
Left ventricular mass index, g/m^2.7^	30.8 (0.2)
Left ventricular systolic function	
Left ventricular ejection fraction, %	63.8 (0.2)
Septal velocity, cm/s	0.786 (0.002)
Left ventricular diastolic function	
Transmitral E/e’ ratio	7.41 (0.04)
Septal e’ velocity, cm/s	1.179 (0.004)
Left atrium anteroposterior diameter index, mm/m	1.823 (0.006)
Transmitral E/A ratio	1.97 (0.01)
Transmitral E deceleration time, ms	170.2 (0.7)
Right ventricular systolic function	
Tricuspid annular plane systolic excursion, cm	2.386 (0.009)
Hemodynamic parameters	
Stroke index, mL/m^2^	35.4 (0.2)
Cardiac index, L/min⋅m^2^	2.35 (0.01)
Central pulse pressure, mm Hg	48.4 (0.2)
Mean arterial pressure, mm Hg	84.9 (0.2)
Heart rate, beats/min	66.8 (0.2)
Total arterial compliance index, mL/mm Hg⋅m^2^	0.76 (0.01)
Systemic vascular resistance index, mm Hg·min⋅m^2^/L	37.9 (0.2)
Vascular	
Carotid intima-media thickness, mm	0.458 (0.001)
Carotid-femoral pulse wave velocity, m/s	6.30 (0.02)

### Steatosis

Steatosis (CAP>275 dB/m) was associated with decreased end-diastolic volume index, greater RWT at end-diastole, lower LVMI, higher E/e’ ratio, lower left atrium anteroposterior diameter index, reduced septal e’ velocity, E/A ratio, and stroke index, as well as higher MAP, heart rate, and SVRI when compared with no steatosis (CAP≤275 dB/m) after adjustment for age, sex, ethnicity, class, and NEET status (Supplemental Table S3, http://links.lww.com/HC9/A223). The associations for end-diastolic volume index, RWT, LVMI, E/e’ ratio, septal e’ velocity, left atrium anteroposterior diameter index, E/A ratio, MAP, and SVRI attenuated after further adjustment for additional confounders and mediators (ie, BMI, alcohol, smoking, MAP, fasting lipids, glucose, insulin, and CRP in addition to age, sex, ethnicity, class, and NEET status) but associations with stroke index and heart rate remained after full adjustment (models 1 and 2, Table 2). There was no evidence of the associations between steatosis and any other cardiovascular measures (models 1 and 2, Table 2). As there is a risk of overadjusting for adiposity by normalizing to BSA, we performed sensitivity analyses, further adjusting for height for BSA-adjusted variables. The findings were similar to those in our main analyses (Supplemental Table S4, http://links.lww.com/HC9/A223). In our sensitivity analyses examining the complete case (non-imputed) data set, all associations between steatosis and cardiovascular variables attenuated to null (models 1–3, Supplemental Table S5, http://links.lww.com/HC9/A223). Our findings were also similar in our sensitivity analyses, further adjusting for physical activity in the subset of patients for which these data were collected (Supplemental Table S6, http://links.lww.com/HC9/A223).

**TABLE 2:** Associations of liver steatosis and fibrosis with cardiovascular structure and function in imputed data set

	Steatosis (N=1970)		Fibrosis (N=1996)	
Cardiovascular variable	Model 1^a^	Model 2^b^	Model 1^a^	Model 2^b^
LV structure				
End-diastolic volume index, mL/m^2^	−2.77 (−4.67, −0.88)	−1.93 (−3.94, 0.07)	1.90 (−1.58, 5.39)	0.99 (−2.39, 4.37)
Relative wall thickness at end-diastole	0.003 (−0.008, 0.015)	0.002 (−0.01, 0.01)	0.01 (−0.01, 0.03)	0.02 (−0.004, 0.04)
Left ventricular mass index, g/m^2.7^	−1.31 (−2.45, −0.18)	−0.97 (−2.17, 0.22)	2.73 (0.80, 4.65)	2.46 (0.56, 4.37)
LV systolic function				
Left ventricular ejection fraction, %	−0.88 (−2.22, 0.47)	−1.20 (−2.55, 0.15)	0.70 (−1.80, 3.20)	0.53 (−1.98, 3.03)
Septal s’ velocity, cm/s	−0.004 (−0.02, 0.01)	−0.002 (−0.020, 0.016)	−0.03 (−0.06, 0.01)	−0.03 (−0.06, 0.01)
LV diastolic function				
E/e’ ratio	0.18 (−0.09, 0.45)	0.12 (−0.15, 0.40)	−0.07 (−0.54, 0.41)	−0.01 (−0.48, 0.46)
Septal e’ velocity, cm/s	−0.04 (−0.07, −0.01)	−0.03 (−0.06, 0.003)	0.05 (−0.01, 0.10)	0.04 (−0.02, 0.09)
Left atrium anteroposterior diameter index, mm/m	−0.02 (−0.06, 0.01)	−0.02 (−0.06, 0.02)	0.06 (−0.01, 0.13)	0.05 (−0.02, 0.12)
Transmitral E/A ratio	−0.05 (−0.15, 0.04)	−0.03 (−0.13, 0.07)	0.35 (0.17, 0.54)	0.32 (0.13, 0.50)
Transmitral E deceleration time, ms	0.18 (−4.89, 5.26)	1.42 (−3.92, 6.77)	7.60 (−2.06, 17.27)	7.11 (−2.52, 16.74)
RV systolic function				
Tricuspid annular plane systolic excursion, cm	−0.06 (−0.13, 0.01)	−0.05 (−0.12, 0.02)	0.16 (0.03, 0.28)	0.14 (0.01, 0.26)
Hemodynamic parameters				
Stroke index, mL/m^2^	−2.21 (−3.63, −0.79)	−1.85 (−3.29, −0.41)	1.52 (−1.07, 4.10)	0.84 (−1.69, 3.36)
Cardiac index, L/min⋅m^2^	−0.01 (−0.11, 0.09)	−0.04 (−0.14, 0.06)	−0.24 (−0.42, −0.06)	−0.23 (−0.41, −0.06)
Central pulse pressure, mm Hg	−1.17 (−2.59, 0.25)	−0.72 (−2.18, 0.74)	−0.27 (−3.10, 2.56)	−0.41 (−3.27, 2.44)
Mean arterial pressure, mm Hg	1.53 (0.08, 2.99)	0.67 (−0.80, 2.14)	−1.32 (−4.09, 1.45)	−0.58 (−3.33, 2.17)
Heart rate, beats/min	3.73 (2.09, 5.37)	2.17 (0.58, 3.75)	−8.63 (−11.78, −5.48)	−7.23 (−10.16, −4.29)
Total arterial compliance index, mL/mm Hg⋅m^2^	−0.05 (−0.15, 0.05)	−0.06 (−0.16, 0.04)	0.03 (−0.13, 0.19)	0.01 (−0.15, 0.17)
Systemic vascular resistance index, mm Hg·min⋅m^2^/L	1.00 (−0.94, 2.93)	1.15 (−0.83, 3.13)	2.95 (−0.38, 6.27)	3.28 (−0.03, 6.59)
Vascular				
Carotid intima-media thickness, mm	−0.0007 (−0.009, 0.008)	0.001 (−0.008, 0.010)	0.024 (0.008, 0.041)	0.024 (0.008, 0.040)
Carotid-femoral pulse wave velocity, m/s	−0.01 (−0.19, 0.17)	−0.05 (−0.23, 0.13)	0.40 (0.06, 0.74)	0.40 (0.06, 0.75)

*Note:* Values reported as mean difference (95% CI).

^a^ Model 1 adjusted for age, sex, ethnicity, class, education/employment/training status, body mass index, alcohol, and smoking.

^b^ Model 2 adjusted for the covariates in Model 1, mean arterial pressure, fasting lipids, glucose, insulin, C-reactive protein; mean arterial pressure excluded in the analyses of mean arterial pressure and systemic vascular resistance.

Abbreviations: LV, left ventricular; RV, right ventricular.

### Fibrosis

Liver fibrosis (liver stiffness ≥7.9 kPa) was associated with greater LVMI, transmitral E/A ratio and TAPSE, greater cIMT and PWV, as well as reduced cardiac index and heart rate when compared with no fibrosis (liver stiffness <7.9 kPa) after adjustment for age, sex, ethnicity, class, and NEET status (Supplemental Table S3, http://links.lww.com/HC9/A223). These associations remained after further adjustment for BMI, alcohol, and smoking (model 1, Table 2), and then additional adjustment for MAP, lipids, glucose, insulin, and CRP (model 2, Table 2). There was no evidence of an association between fibrosis and total arterial compliance, nor with any other cardiovascular measures in any of our models (models 1–2, Table 2). Our sensitivity analyses, further adjusting for height in our BSA-indexed variables, estimated similar associations to those in our main analyses (Supplemental Table S4, http://links.lww.com/HC9/A223). Associations between fibrosis and cardiovascular measures were also similar in sensitivity analyses of our complete case (non-imputed) data (models 1–3, Supplemental Table S5, http://links.lww.com/HC9/A223), as well as in our sensitivity analyses further adjusting for physical activity in the subset of participants who had these data available (Supplemental Table S6, http://links.lww.com/HC9/A223).

## DISCUSSION

In this general population cohort, liver fibrosis measured by transient elastography was associated with greater LVMI, transmitral E/A ratio, and TAPSE, as well as lower cardiac index and heart rate even after full adjustment for cardiometabolic risk factors (ie, age, sex, ethnicity, class, NEET status, BMI, alcohol, smoking, MAP, and serum biomarkers). Liver fibrosis was also associated with increased cIMT and carotid-femoral PWV. Fibrosis was not associated with measures of LV systolic function (ejection fraction or s’ velocity) nor with measures of LV diastolic function (E/e’ ratio, e’ velocity, E deceleration time or left atrial diameter index) aside from E/A ratio. Furthermore, fibrosis was associated with only small differences in LVMI, E/A ratio, and TAPSE (an indicator of RV systolic function). The decrease in the cardiac index is likely a result of the decrease in heart rate observed previously in liver fibrosis,^29^ and given the known load dependence of LVMI, E/A ratio, and TAPSE, these differences may also be linked to the lower heart rate and possibly consequently increased preload. Liver steatosis was associated with a decrease in both stroke index and an increase in heart rate, but all other associations between steatosis and cardiovascular measures attenuated to the null in our fully adjusted model.

Few studies have previously examined the associations between NAFLD and echocardiography measurements while adjusting for confounding in a general population sample. Li and colleagues used data from the prospective Bogalusa Heart Study and Young Finns Study cohorts, and they reported a positive association between fatty liver index score^30^ and an increase in LVMI in a young adult population after adjusting for age, sex, ethnicity, smoking, alcohol, physical activity, blood pressure, lipids, glucose, insulin resistance, CRP, use of antihypertensive(s), and use of cholesterol-lowering medication.^6^ In 2016, Mantovani and colleagues summarized the evidence of association between NAFLD and LV diastolic dysfunction, greater LV filling pressure, left atrial volume, global longitudinal strain, and other cardiac measurements after adjustment for cardiometabolic risks, although the included samples ranged from 38 to 2713 (18 out of 20 with fewer than 500 participants).^7^ More recently, VanWagner and colleagues also reported changes in echocardiographic LV measurements (increased LV mass, RWT, E/A ratio, and cardiac index; decreased LV ejection fraction and E/A ratio) associated with CT-defined steatosis.^11^ Notably, we did not find evidence of any association between NAFLD and diastolic dysfunction in our present work. Other recent studies reported associations between both steatosis and fibrosis with other measures of LV diastolic dysfunction (E/e’ ratio, E/A ratio, and isovolumetric relaxation time) 8,9 and LV systolic dysfunction (ejection fraction, peak longitudinal strain, peak circumferential strain, and peak radial strain)^8^ after adjustment for established cardiovascular covariates. Although de Freitas Diniz et al^10^ found fibrosis, but not steatosis, to be associated with reduced LV ejection fraction. Compared with the present work, these previous reports include smaller samples, older populations, more severe NAFLD, and/or only patients with concurrent diseases such as type 2 diabetes^8^ and obesity requiring bariatric surgery.^10^

Previous studies reported an increase in cIMT and PWV (ie, measures of subclinical atherosclerosis) in the presence of NAFLD, independent of the shared cardiometabolic risks between atherosclerosis and NAFLD, but many reports describe case-control studies in hospital patients with NAFLD compared with hospital-based controls and most studies use ultrasound-defined steatosis and composite laboratory biomarker-based fibrosis scoring. This includes a recent systematic review and meta-analysis by Wong et al^5^, which reports NAFLD is associated with an increase in cIMT. Of the 44 studies (total n=41,189) included, 13 studies used logistic regression to estimate associations, whereas the remaining 21 studies compared mean differences in cIMT.^5^ The pooled OR (95% CI) of an increased cIMT in the presence of NAFLD was 2.00 (1.56, 2.56) when including all studies, but the OR (95% CI) was 1.17 (0.49, 1.85) after excluding studies which did not adjust for any covariates.^5^ However, 6 of the studies with adjusted analyses only adjusted for age, or age and sex.^5^ Furthermore, only 5 of these studies used liver biopsy (n=938) to assess for NAFLD, whereas 2 studies used a mix of either ultrasound or liver biopsy (n=245 with no breakdown of who had undergone biopsy), only 1 study of government officials used Fibroscan transient elastography (n=131), and all others used ultrasonography, CT or magnetic resonance spectroscopy alone to assess for NAFLD.^5^ Liver transient elastography, the method in the present work, is the standard non-invasive measure of steatosis and fibrosis, with greater sensitivity than both ultrasonography and composite fibrosis scoring.^26^,^27^

A systematic review by Zhou et al^31^ identified 2 studies (total n=5909) investigating NAFLD and PWV (ie, another indicator of subclinical atherosclerosis). A more recent report of a general population cohort of 3433 older adults (≥40 y) in Shanghai also estimated a positive association between ultrasonography-defined NAFLD (includes steatosis and fibrosis) and increased cIMT after adjustment for confounding, but they did not find evidence of an association between fibrosis score as estimated by laboratory blood tests with cIMT.^13^ In this study, Xin et al^13^ also estimated a positive association between NAFLD and PWV, as well as between fibrosis score and PWV, after adjustment for age, sex, education, smoking, alcohol, physical activity, obesity, hypertension, type 2 diabetes, and dyslipidemia. The authors did not examine associations between steatosis alone (without fibrosis) and cIMT or PWV.^13^ Unlike the general population setting and Fibroscan assessments of NAFLD used in the present study, these 2 studies were conducted in hospital settings and relied on ultrasound-defined NAFLD.

More recent studies also report associations between fibrosis and atherosclerosis measures cIMT and PWV. A case-control study of fibrosis (ie, not including steatosis alone) in adults aged 30–70 years (n=213 with fibrosis) by Taharboucht et al^32^ also reported greater cIMT and greater carotid-femoral PWV in the presence of fibrosis, even after the adjustment for age, sex, smoking, abdominal obesity, overall obesity, hypertension, dyslipidemia, GGT, ALT, insulin resistance, and CRP. These findings by Taharboucht et al^32^ are similar to those reported in the present work.

Additional recent reports have demonstrated positive associations between NAFLD and fibrosis with increased cIMT and PWV, but these were in older populations compared with the present study (age>40 y), relying on less sensitive modalities to define NAFLD, including smaller case-control samples and/or did not adjust for confounding.^33^–^37^ A review of by Karjoo^12^ found similar limitations in pediatric studies of NAFLD and atherosclerosis.

There are numerous potential mechanisms that may help to explain the associations between fibrosis and changes in cardiovascular structure and function reported in the present work. The progression of NAFL steatosis to NASH fibrosis is characterized by chronic inflammation, gut microbiota dysbiosis, worsening insulin resistance, and systemic release of adipokines. These extrahepatic changes, not often present in steatosis alone, have themselves been associated with an increased risk of atherosclerosis and changes in cardiac structure and function.^2^,^38^

To the best of our knowledge, our present work is the only study to investigate associations of transient elastography-defined liver steatosis and fibrosis with cIMT, PWV, and total arterial compliance with multiple-adjustment for confounding. The strengths of our work include our large sample, comprehensive adjustment for cardiometabolic confounding, prospective data collection from participants’ birth onward, and measuring steatosis and fibrosis using FibroScan transient elastography, which is the standard non-invasive method for staging fibrosis and grading steatosis.^1^,^26^,^27^,^39^ Our study also has limitations. NAFLD and cardiovascular measures were assessed concurrently, and there may be a lag in the potential effect of NAFLD on the heart, or effects may take time to accumulate. Cutoff values for liver stiffness measurements and controlled attenuation parameters are validated in patients with histologically proven NAFLD, who tend to be in the fifth and sixth decade of life.^26^,^27^ In addition, only 38 participants in the present cohort have liver fibrosis, but a report by Abeysekera et al^15^ in this same ALSPAC cohort and a report by Zhang et al^40^ in the National Health and Nutrition Examination Survey in the US are the only 2 studies to date in which disease burden has been described in this age group. The National Health and Nutrition Examination Survey data^40^ estimated higher rates of NAFLD in adults aged 20–29 years when compared with the ALSPAC cohort at age 24 years. Our cohort may underestimate prevalence of NAFLD in populations with greater obesity prevalence; however, the prevalence of obesity in the National Health and Nutrition Examination Survey is greater than that found in ALSPAC, and rates of obesity in the south west of the UK is similar to that of the UK average.^41^ Lastly, only 681/2047 (33.3%) participants had accelerometer data available, but sensitivity analyses in this subset further adjusting for physical activity (in addition to the covariates in our fully adjusted model 2) showed similar findings as those in our main analyses (Supplemental Table S6, http://links.lww.com/HC9/A223).

To conclude, our findings suggest that NAFLD-related fibrosis, but not steatosis, is associated with changes in cardiac structure and function as measured by echocardiography and with measures of subclinical atherosclerosis, even after adjustment for established cardiometabolic risk factors. We did not, however, find any evidence of association between fibrosis and LV systolic function and observed differences were small in comparison to clinical thresholds for the diagnosis of LV diastolic dysfunction or RV systolic dysfunction. Further follow-up of this and similar cohorts will help determine whether cardiovascular health worsens over time in those with steatosis alone, including those who progress to fibrosis. However, our findings are consistent with clinical practice guidance identifying fibrosis as the most prognostic indicator in NAFLD when compared with age, sex, obesity, smoking, diabetes, hypertension, concurrent cardiovascular disease, dyslipidemia, or liver enzymes.^1^

Given that the leading cause of mortality in NAFLD is cardiovascular disease, our demonstration of cardiovascular changes in young, otherwise “healthy” adults in a general population cohort is concerning and suggests clinicians may soon see patients present earlier with NASH cirrhosis and associated cardiovascular complications. More research is required to investigate whether the treatment of NASH improves cardiovascular outcomes.

## Supplementary Material
