# Supplementary material for: Non-alcoholic fatty liver and fibrosis is associated with cardiovascular structure and function in young adults
Source: Hepatol Commun. 2023 Mar 30;7(4):e0087. doi: 10.1097/HC9.0000000000000087 (PMC10069841; doi:10.1097/HC9.0000000000000087)
Supplement: Supplementary file 1 [file hc9-7-e0087-s001.docx]

# Supporting Information

## Supplementary Tables

## **Table S1.** Distribution of observed (non-imputed) and imputed characteristics (N = 2,047)

| **Characteristic** | **Percentage of data imputed** | **Mean (SE) or proportion in observed data** | **Mean (SE) or proportion in imputed data** |
| --- | --- | --- | --- |
| **Exposures** |  |  |  |
| Controlled attenuation parameter (CAP), db/m | Not imputed | 208.1 (1.2) | 208.1 (1.2) |
| Steatosis  CAP ≤ 275 db/m  CAP > 275 db/m  Missing | Not imputed | 86%  10%  4% | 86%  10%  4% |
| Liver stiffness, kPa | Not imputed | 4.75 (0.03) | 4.75 (0.03) |
| Fibrosis  Liver stiffness < 7.9 kPa  Liver stiffness ≥ 7.9 kPa  Missing | Not imputed | 96%  2%  2% | 96%  2%  2% |
| **Outcomes** |  |  |  |
| *Left ventricular structure* |  |  |  |
| End-diastolic volume index, mL/m^2^ | 19.8 | 55.9 (0.2) | 55.7 (0.3) |
| Relative wall thickness at end-diastole | 19.7 | 0.365 (0.001) | 0.366 (0.002) |
| Left ventricular mass index, g/m^2.7^ | 20.1 | 30.4 (0.2) | 30.8 (0.2) |
| *Left ventricular systolic function* |  |  |  |
| Left ventricular ejection fraction, % | 22.1 | 63.9 (0.2) | 63.8 (0.2) |
| Septal s’ velocity, cm/s | 11.9 | 0.786 (0.002) | 0.786 (0.002) |
| *Left ventricular diastolic function* |  |  |  |
| Transmitral E/e’ ratio | 15.6 | 7.41 (0.03) | 7.41 (0.04) |
| Septal e’ velocity, cm/s | 11.8 | 1.180 (0.004) | 1.179 (0.004) |
| Left atrium anteroposterior  diameter index, mm/m | 11.5 | 1.818 (0.006) | 1.823 (0.006) |
| Transmitral E/A ratio | 13.1 | 1.98 (0.01) | 1.97 (0.01) |
| Transmitral E deceleration time, ms | 13.0 | 170.3 (0.7) | 170.2 (0.7) |
| *Right ventricular systolic function* |  |  |  |
| Tricuspid annular plane systolic excursion, cm | 19.5 | 2.39 (0.01) | 2.39 (0.01) |
| *Hemodynamic parameters* |  |  |  |
| Stroke index, mL/m^2^ | 22.5 | 35.6 (0.2) | 35.4 (0.2) |
| Cardiac index, L/min⋅m^2^ | 22.8 | 2.3 (0.01) | 2.35 (0.01) |
| Central pulse pressure, mmHg | 6.7 | 48.4 (0.2) | 48.4 (0.2) |
| Mean arterial pressure, mmHg | 6.7 | 84.7 (0.2) | 84.9 (0.2) |
| Heart rate, beats/min | 0.3 | 66.8 (0.2) | 66.8 (0.2) |
| Total arterial compliance index, mL/mmHg⋅m^2^ | 27.1 | 0.77 (0.01) | 0.76 (0.01) |
| Systemic vascular resistance index, mmHg·min⋅m^2^/L | 27.1 | 37.6 (0.2) | 37.9 (0.2) |
| *Vascular* |  |  |  |
| Carotid intima-media thickness, mm | 14.8 | 0.458 (0.001) | 0.458 (0.001) |
| Carotid-femoral pulse wave velocity, m/s | 6.5 | 6.30 (0.02) | 6.30 (0.02) |
| **Covariates** |  |  |  |
| Age, years | 0 | 24.36 (0.01) | 24.36 (0.01) |
| Female sex | 0 | 36.2% | 36.2% |
| Non-White ethnicity | 7.6 | 2% | 3% |
| Highest parental social class  I  II  III (non-manual)  III (manual)  IV  V | 17.6 | 20%  48%  25%  6%  2%  0.1% | 19%  48%  24%  6%  2%  1% |
| Not in education, employment or training | 2.8 | 8% | 8% |
| Body mass index, kg/m^2^ | 1.0 | 24.6 (0.1) | 24.7 (0.1) |
| Alcohol units per day of drinking  Zero to two  Three to six  Seven or more | 2.3 | 29%  52%  19% | 29%  52%  19% |
| Smoking status  Never smoker  Ex-smoker  Current smoker | 1.1 | 42%  36%  22% | 42%  36%  22% |
| Fasting triglycerides, mmol/L | 19.1 | 0.95 (0.01) | 0.95 (0.01) |
| Fasting total cholesterol, mmol/L | 19.1 | 4.38 (0.02) | 4.39 (0.02) |
| Fasting HDL cholesterol, mmol/L | 19.1 | 1.55 (0.01) | 1.54 (0.01) |
| Fasting glucose, mmol/L | 19.1 | 5.3 (0.01) | 5.3 (0.01) |
| Fasting insulin, mIU/L | 19.1 | 9.8 (0.2) | 10.0 (0.2) |
| C-reactive protein, mg/L | 26.5 | 2.3 (0.2) | 2.2 (0.2) |
| **Auxiliary imputation variables** |  |  |  |
| Fasting triglycerides at age 17, mmol/L | 40.8 | 0.82 (0.01) | 0.83 (0.01) |
| Fasting total cholesterol at age 17, mmol/L | 40.8 | 3.81 (0.02) | 3.83 (0.02) |
| Fasting glucose at age 17, mmol/L | 40.8 | 5.00 (0.01) | 5.00 (0.01) |
| Fasting insulin at age 17, mIU/L | 41.8 | 8.2 (0.2) | 8.3 (0.2) |
| Alanine transaminase at age 17, IU/L | 41.3 | 17.3 (0.3) | 17.4 (0.3) |
| Gamma glutamyl transferase at age 17, IU/L | 41.3 | 18.0 (0.3) | 18.3 (0.3) |
| C-reactive protein at age 17, mg/L | 40.8 | 1.6 (0.2) | 1.7 (0.5) |
| Peripheral systolic blood pressure at age 24, mmHg | 0.3 | 115.3 (0.3) | 115.3 (0.3) |
| Peripheral diastolic blood pressure at age 24, mmHg | 0.3 | 66.5 (0.2) | 66.5 (0.2) |
| Fasting LDL cholesterol at age 24, mmol/L | 19.1 | 2.40 (0.02) | 2.42 (0.02) |
| Alanine transaminase at age 24, IU/L | 19.2 | 26.2 (0.6) | 26.3 (0.6) |
| Gamma glutamyl transferase at age 24, IU/L | 19.1 | 19.4 (0.5) | 19.5 (0.5) |

Abbreviations: SE, standard error; HDL, high-density lipoprotein; LDL, low-density liproprotein

## **Table S2.** Variables used in imputation model

| **Characteristic** | **Type of variable** | **Regression model to predict missingness** |
| --- | --- | --- |
| **Exposures** |  |  |
| Liver steatosis (controlled attenuation parameter) | Continuous | Not imputed |
| Liver fibrosis | Continuous | Not imputed |
| **Outcomes** |  |  |
| *Left ventricular structure* |  |  |
| End-diastolic volume indexed to BSA | Continuous | Linear regression |
| Relative wall thickness at end-diastole | Continuous | Linear regression |
| Left ventricular mass indexed to height^2.7^ | Continuous | Linear regression |
| *Left ventricular systolic function* |  |  |
| Left ventricular ejection fraction | Continuous | Linear regression |
| Septal s’ velocity | Continuous | Linear regression |
| *Left ventricular diastolic function* |  |  |
| Transmitral E/e’ ratio | Continuous | Linear regression |
| Septal e’ velocity | Continuous | Linear regression |
| Left atrium anteroposterior diameter indexed to height | Continuous | Linear regression |
| Transmitral E/A ratio | Continuous | Linear regression |
| Transmitral E deceleration time | Continuous | Linear regression |
| *Right ventricular systolic function* |  |  |
| Tricuspid annular plane systolic excursion | Continuous | Linear regression |
| *Hemodynamic parameters* |  |  |
| Stroke index | Continuous | Linear regression |
| Cardiac index | Continuous | Linear regression |
| Central pulse pressure | Continuous | Linear regression |
| Mean arterial pressure | Continuous | Linear regression |
| Heart rate | Continuous | Linear regression |
| Total arterial compliance indexed to BSA | Continuous | Linear regression |
| Systemic vascular resistance indexed to BSA | Continuous | Linear regression |
| *Vascular* |  |  |
| Carotid intima-media thickness | Continuous | Linear regression |
| Carotid-femoral pulse wave velocity | Continuous | Linear regression |
| **Covariates** |  |  |
| Age | Continuous | Linear regression |
| Sex | Categorical (2) | Logistic regression |
| Ethnicity | Categorical (2) | Logistic regression |
| Highest parental social class | Categorical (6) | Logistic regression |
| Not in education, employment or training | Categorical (2) | Logistic regression |
| Body mass index | Continuous | Linear regression |
| Alcohol units per day of drinking | Categorical (3) | Logistic regression |
| Smoking status | Categorical (3) | Logistic regression |
| Triglycerides | Continuous | Linear regression |
| Total cholesterol | Continuous | Linear regression |
| HDL cholesterol | Continuous | Linear regression |
| Glucose | Continuous | Linear regression |
| Insulin | Continuous | Linear regression |
| C-reactive protein | Continuous | Linear regression |
| **Auxiliary imputation variables** |  |  |
| Triglycerides at age 17 | Continuous | Linear regression |
| Total cholesterol at age 17 | Continuous | Linear regression |
| Glucose at age 17 | Continuous | Linear regression |
| Insulin at age 17 | Continuous | Linear regression |
| Alanine transaminase at age 17 | Continuous | Linear regression |
| Gamma glutamyl transferase at age 17 | Continuous | Linear regression |
| C-reactive protein at age 17 | Continuous | Linear regression |
| Peripheral systolic blood pressure at age 24 | Continuous | Linear regression |
| Peripheral diastolic blood pressure at age 24 | Continuous | Linear regression |
| LDL cholesterol at age 24 | Continuous | Linear regression |
| Alanine transaminase at age 24 | Continuous | Linear regression |
| Gamma glutamyl transferase at age 24 | Continuous | Linear regression |

Abbreviations: BSA, body surface area; HDL, high-density lipoprotein; LDL, low-density lipoprotein

## **Table S3.** Associations of liver steatosis and fibrosis with cardiovascular structure and function with models separately adjusting for demographics (model 1) and confounders or potential mediators (models 2 and 3)

| **Cardiovascular imaging measure** | **Steatosis (N = 1,970)** | | | **Fibrosis (N = 1,996)** | | |
| --- | --- | --- | --- | --- | --- | --- |
|  | **Model 1^a^** | **Model 2^b^** | **Model 3^c^** | **Model 1^a^** | **Model 2^b^** | **Model 3^c^** |
| ***LV structure*** |  |  |  |  |  |  |
| End-diastolic volume index, mL/m^2^ | -4.05 (-5.82, -2.28) | -2.77 (-4.67, -0.88) | -1.93 (-3.94, 0.07) | 1.79 (-1.71, 5.28) | 1.90 (-1.58, 5.39) | 0.99 (-2.39, 4.37) |
| Relative wall thickness at end-diastole | 0.02 (0.01, 0.03) | 0.003 (-0.008, 0.015) | 0.002 (-0.01, 0.01) | 0.02 (-0.01, 0.04) | 0.01 (-0.01, 0.03) | 0.02 (-0.004, 0.04) |
| Left ventricular mass index, g/m^2.7^ | 4.25 (3.11, 5.40) | -1.31 (-2.45, -0.18) | -0.97 (-2.17, 0.22) | 2.98 (0.74, 5.23) | 2.73 (0.80, 4.65) | 2.46 (0.56, 4.37) |
| ***LV systolic function*** |  |  |  |  |  |  |
| Left ventricular ejection fraction, % | -0.60 (-1.86, 0.66) | -0.88 (-2.22, 0.47) | -1.20 (-2.55, 0.15) | 0.77 (-1.72, 3.26) | 0.70 (-1.80, 3.20) | 0.53 (-1.98, 3.03) |
| Septal s’ velocity, cm/s | -0.01 (-0.02, 0.01) | -0.004 (-0.02, 0.01) | -0.002 (-0.020, 0.016) | -0.03 (-0.06, 0.01) | -0.03 (-0.06, 0.01) | -0.03 (-0.06, 0.01) |
| ***LV diastolic function*** |  |  |  |  |  |  |
| Transmitral E/e’ ratio | 0.65 (0.40, 0.90) | 0.18 (-0.09, 0.45) | 0.12 (-0.15, 0.40) | -0.04 (-0.53, 0.44) | -0.07 (-0.54, 0.41) | -0.01 (-0.48, 0.46) |
| Septal e’ velocity, cm/s | -0.11 (-0.14, -0.08) | -0.04 (-0.07, -0.01) | -0.03 (-0.06, 0.003) | 0.04 (-0.01, 0.10) | 0.05 (-0.01, 0.10) | 0.04 (-0.02, 0.09) |
| Left atrium anteroposterior diameter index, mm/m | 0.19 (0.15, 0.23) | -0.02 (-0.06, 0.01) | -0.02 (-0.06, 0.02) | 0.07 (-0.01, 0.15) | 0.06 (-0.01, 0.13) | 0.05 (-0.02, 0.12) |
| Transmitral E/A ratio | -0.17 (-0.26, -0.08) | -0.05 (-0.15, 0.04) | -0.03 (-0.13, 0.07) | 0.35 (0.16, 0.54) | 0.35 (0.17, 0.54) | 0.32 (0.13, 0.50) |
| Transmitral E deceleration time, ms | -0.28 (-4.80, 4.25) | 0.18 (-4.89, 5.26) | 1.42 (-3.92, 6.77) | 7.92 (-1.74, 17.59) | 7.60 (-2.06, 17.27) | 7.11 (-2.52, 16.74) |
| ***RV systolic function*** |  |  |  |  |  |  |
| Tricuspid annular plane systolic excursion, cm | 0.01 (-0.06, 0.08) | -0.06 (-0.13, 0.01) | -0.05 (-0.12, 0.02) | 0.17 (0.04, 0.30) | 0.16 (0.03, 0.28) | 0.14 (0.01, 0.26) |
| ***Hemodynamic parameters*** |  |  |  |  |  |  |
| Stroke index, mL/m^2^ | -2.87 (-4.23, -1.51) | -2.21 (-3.63, -0.79) | -1.85 (-3.29, -0.41) | 1.48 (-1.10, 4.06) | 1.52 (-1.07, 4.10) | 0.84 (-1.69, 3.36) |
| Cardiac index, L/min⋅m^2^ | -0.01 (-0.10, 0.09) | -0.01 (-0.11, 0.09) | -0.04 (-0.14, 0.06) | -0.24 (-0.42, -0.06) | -0.24 (-0.42, -0.06) | -0.23 (-0.41, -0.06) |
| Central pulse pressure, mmHg | 0.96 (-0.29, 2.22) | -1.17 (-2.59, 0.25) | -0.72 (-2.18, 0.74) | -0.13 (-2.97, 2.71) | -0.27 (-3.10, 2.56) | -0.41 (-3.27, 2.44) |
| Mean arterial pressure, mmHg | 6.81 (5.45, 8.17) | 1.53 (0.08, 2.99) | 0.67 (-0.80, 2.14) | -1.07 (-4.07, 1.93) | -1.32 (-4.09, 1.45) | -0.58 (-3.33, 2.17) |
| Heart rate, beats/min | 5.13 (3.68, 6.58) | 3.73 (2.09, 5.37) | 2.17 (0.58, 3.75) | -8.67 (-11.86, -5.48) | -8.63 (-11.78, -5.48) | -7.23 (-10.16, -4.29) |
| Total arterial compliance index, mL/mmHg⋅m^2^ | -0.08 (-0.17, 0.01) | -0.05 (-0.15, 0.05) | -0.06 (-0.16, 0.04) | 0.02 (-0.14, 0.18) | 0.03 (-0.13, 0.19) | 0.01 (-0.15, 0.17) |
| Systemic vascular resistance index, mmHg·min⋅m^2^/L | 3.41 (1.58, 5.23) | 1.00 (-0.94, 2.93) | 1.15 (-0.83, 3.13) | 3.13 (-0.24, 6.50) | 2.95 (-0.38, 6.27) | 3.28 (-0.03, 6.59) |
| ***Vascular*** |  |  |  |  |  |  |
| Carotid intima-media thickness, mm | 0.004 (-0.003, 0.012) | -0.0007 (-0.009, 0.008) | 0.001 (-0.008, 0.010) | 0.025 (0.009, 0.041) | 0.024 (0.008, 0.041) | 0.024 (0.008, 0.040) |
| Carotid-femoral pulse wave velocity, m/s | 0.09 (-0.07, 0.25) | -0.01 (-0.19, 0.17) | -0.05 (-0.23, 0.13) | 0.41 (0.06, 0.75) | 0.40 (0.06, 0.74) | 0.40 (0.06, 0.75) |

Abbreviations: LV, left ventricular; RV, right ventricular

Note: Values reported as mean difference (95% confidence interval)

^a^Model 1 adjusted for age, sex, ethnicity, class, education/employment/training status

^b^Model 2 adjusted for the covariates in Model 1, body mass index, alcohol, smoking

^c^Model 3 adjusted for the covariates in Model 2, mean arterial pressure, fasting lipids, glucose, insulin, C-reactive protein; mean arterial pressure excluded in analyses of mean arterial pressure and systemic vascular resistance

## **Table S4.** Associations of liver steatosis and fibrosis with cardiovascular structure and function further adjusting for height in body surface area-indexed outcomes

| **Cardiovascular variable** | **Steatosis** | | **Fibrosis** | |
| --- | --- | --- | --- | --- |
|  | **Model 3^a^ without adjustment for height** | **Model 3 with further adjustment for height** | **Model 3^a^ without adjustment for height** | **Model 3 with further adjustment for height** |
| ***Left ventricular structure*** |  |  |  |  |
| End-diastolic volume index, mL/m^2^ | -1.93 (-3.94, 0.07) | -1.91 (-3.93, 0.10) | 0.99 (-2.39, 4.37) | 1.01 (-2.38, 4.40) |
| ***Hemodynamic parameters*** |  |  |  |  |
| Stroke index, mL/m^2^ | -1.85 (-3.29, -0.41) | -1.76 (-3.22, -0.30) | 0.84 (-1.69, 3.36) | 0.89 (-1.64, 3.41) |
| Cardiac index, L/min⋅m^2^ | -0.04 (-0.14, 0.06) | -0.03 (-0.13, 0.07) | -0.23 (-0.41, -0.06) | -0.23 (-0.41, -0.05) |
| Total arterial compliance index, mL/mmHg⋅m^2^ | -0.06 (-0.16, 0.04) | -0.06 (-0.16, 0.04) | 0.01 (-0.15, 0.17) | 0.01 (-0.15, 0.17) |
| Systemic vascular resistance index, mmHg·min⋅m^2^/L | 1.15 (-0.83, 3.13) | 0.97 (-1.03, 2.96) | 3.28 (-0.03, 6.59) | 3.19 (-0.12, 6.50) |

Note: Values are reported as mean difference (95% confidence interval)

^a^Model 3 adjusted for age, sex, ethnicity, parents’ highest social class and young person’s education/employment/training status, body mass index, physical activity alcohol, smoking, mean arterial pressure, fasting lipids, glucose, insulin, C-reactive protein; mean arterial pressure excluded in analyses of mean arterial pressure and systemic vascular resistance

## **Table S5.** Complete case analyses of associations of liver steatosis and fibrosis with cardiovascular structure and function

| **Cardiovascular imaging measure** | **Steatosis** | | | **Fibrosis** | | |
| --- | --- | --- | --- | --- | --- | --- |
|  | **Model 1^a^** | **Model 2^b^** | **Model 3^c^** | **Model 1^a^** | **Model 2^b^** | **Model 3^c^** |
| ***Left ventricular structure*** |  |  |  |  |  |  |
| End-diastolic volume index, mL/m^2^ | -3.07 (-5.06, -1.08) | -2.00 (-4.19, 0.18) | 0.07 (-3.99, 4.12) | 1.33 (-2.41, 5.06) | 1.94 (-1.86, 5.74) | 1.43 (-2.92, 5.78) |
| Relative wall thickness at end-diastole | 0.01 (0.002, 0.03) | 0.003 (-0.01, 0.02) | -0.01 (-0.03, 0.02) | 0.02 (-0.004, 0.04) | 0.01 (-0.01, 0.04) | 0.02 (-0.01, 0.05) |
| Left ventricular mass index, g/m^2.7^ | 3.90 (2.55, 5.25) | -0.85 (-2.17, 0.47) | -0.74 (-3.06, 1.58) | 2.62 (0.09, 5.14) | 2.93 (0.67, 5.20) | 2.90 (0.14, 5.65) |
| ***Left ventricular systolic function*** |  |  |  |  |  |  |
| Left ventricular ejection fraction, % | -1.13 (-2.55, 0.30) | -1.48 (-3.05, 0.10) | -0.85 (-4.12, 2.41) | 0.92 (-1.74, 3.57) | 0.73 (-1.99, 3.45) | -0.15 (-3.39, 3.09) |
| Septal s’ velocity, cm/s | -0.01 (-0.02, 0.01) | -0.01 (-0.03, 0.01) | 0.0005 (-0.03, 0.04) | -0.03 (-0.06, 0.01) | -0.03 (-0.06, 0.01) | -0.04 (-0.08, 0.01) |
| ***Left ventricular diastolic function*** |  |  |  |  |  |  |
| Transmitral E/e’ ratio | 0.60 (0.33, 0.86) | 0.16 (-0.13, 0.46) | -0.08 (-0.67, 0.50) | 0.08 (-0.47, 0.63) | 0.08 (-0.47, 0.63) | 0.27 (-0.37, 0.91) |
| Septal e’ velocity, cm/s | -0.11 (-0.14, -0.08) | -0.05 (-0.08, -0.01) | -0.02 (-0.08, 0.05) | 0.05 (-0.02, 0.11) | 0.04 (-0.03, 0.10) | -0.01 (-0.08, 0.06) |
| Left atrium anteroposterior diameter index, mm/m | 0.19 (0.15, 0.23) | -0.01 (-0.05, 0.03) | -0.02 (-0.09, 0.05) | 0.06 (-0.03, 0.15) | 0.06 (-0.02, 0.13) | 0.05 (-0.04, 0.13) |
| Transmitral E/A ratio | -0.17 (-0.27, -0.06) | -0.05 (-0.17, 0.07) | 0.03 (-0.07, 0.14) | 0.27 (0.05, 0.49) | 0.28 (0.06, 0.50) | 0.21 (-0.04, 0.46) |
| Transmitral E deceleration time, ms | 1.53 (-3.64, 6.71) | 2.75 (-3.11, 8.60) | -2.34 (-12.04, 7.37) | 8.84 (-1.97, 19.65) | 9.79 (-1.25, 20.84) | 8.76 (-3.83, 21.35) |
| ***Right ventricular systolic function*** |  |  |  |  |  |  |
| Tricuspid annular plane systolic excursion, cm | -0.003 (-0.07, 0.07) | -0.07 (-0.15, 0.01) | 0.03 (-0.11, 0.16) | 0.15 (0.02, 0.29) | 0.18 (0.04, 0.32) | 0.14 (-0.02, 0.30) |
| ***Hemodynamic parameters*** |  |  |  |  |  |  |
| Stroke index, mL/m^2^ | -2.68 (-4.17, -1.19) | -1.96 (-3.60, -0.33) | -0.89 (-4.14, 2.35) | 1.31 (-1.41, 4.02) | 1.57 (-1.19, 4.33) | 0.84 (-2.35, 4.03) |
| Cardiac index, L/min⋅m^2^ | 0.02 (-0.09, 0.13) | 0.05 (-0.07, 0.17) | 0.01 (-0.09, 0.11) | -0.30 (-0.50, -0.10) | -0.29 (-0.50, -0.09) | -0.20 (-0.43, 0.04) |
| Central pulse pressure, mmHg | 1.24 (-0.12, 2.60) | -1.29 (-2.82, 0.25) | -1.39 (-4.12, 1.34) | -1.20 (-4.19, 1.79) | -1.48 (-4.49, 1.53) | -1.12 (-4.60, 2.35) |
| Mean arterial pressure, mmHg | 6.98 (5.47, 8.49) | 1.63 (0.01, 3.25) | 0.83 (-2.49, 4.16) | -0.51 (-3.88, 2.86) | -0.60 (-3.77, 2.56) | -0.40 (-3.94, 3.15) |
| Heart rate, beats/min | 5.57 (4.01, 7.14) | 4.40 (2.61, 6.19) | -0.02 (-0.10, 0.07) | -9.31 (-12.89, -5.74) | -9.36 (-12.97, -5.74) | -5.67 (-9.52,  -1.81) |
| Total arterial compliance index, mL/mmHg⋅m^2^ | -0.11 (-0.22, 0.001) | -0.08 (-0.20, 0.04) | 0.76 (-2.29, 3.80) | 0.05 (-0.14, 0.24) | 0.06 (-0.14, 0.25) | 0.02 (-0.25, 0.28) |
| Systemic vascular resistance index, mmHg·min⋅m^2^/L | 2.83 (0.75, 4.91) | 0.48 (-1.78, 2.74) | -0.06 (-4.05, 3.92) | 4.38 (0.60, 8.16) | 4.31 (0.50, 8.12) | 2.78 (-1.57, 7.13) |
| ***Vascular*** |  |  |  |  |  |  |
| Carotid intima-media thickness, mm | -0.0002 (-0.008, 0.008) | -0.003 (-0.01, 0.006) | -0.004 (-0.02, 0.01) | 0.02 (0.01, 0.04) | 0.02 (0.004, 0.04) | 0.02 (0.005, 0.04) |
| Carotid-femoral pulse wave velocity, m/s | 0.17 (-0.01, 0.35) | 0.04 (-0.16, 0.25) | -0.10 (-0.50, 0.29) | 0.47 (0.09, 0.86) | 0.48 (0.09, 0.87) | 0.48 (0.02, 0.93) |

Note: Values reported as mean difference (95% confidence interval)

^a^Model 1 adjusted for age, sex, ethnicity, class, education/employment/training status

^b^Model 2 adjusted for the covariates in Model 1, body mass index, alcohol, smoking

^c^Model 3 adjusted for the covariates in Model 2, mean arterial pressure, fasting lipids, glucose, insulin, C-reactive protein; mean arterial pressure was excluded in analyses of mean arterial pressure and systemic vascular resistance

## **Table S6.** Associations of liver steatosis and fibrosis with cardiovascular structure and function with additional covariate physical activity in observed dataset

| **Cardiovascular imaging measure** | **Steatosis** | | **Fibrosis** | |
| --- | --- | --- | --- | --- |
|  | **Model 3^a^ without adjustment for physical activity** | **Model 3^a^ with further adjustment for physical activity** | **Model 3^a^ without adjustment for physical activity** | **Model 3^a^ with further adjustment for physical activity** |
| ***Left ventricular structure*** |  |  |  |  |
| End-diastolic volume index, mL/m^2^ | 0.07 (-3.99, 4.12) | 0.07 (-3.99, 4.12) | 1.43 (-2.92, 5.78) | 2.43 (-4.88, 9.74) |
| Relative wall thickness at end-diastole | -0.01 (-0.03, 0.02) | -0.01 (-0.03, 0.02) | 0.02 (-0.01, 0.05) | 0.02 (-0.02, 0.06) |
| Left ventricular mass index, g/m^2.7^ | -0.74 (-3.06, 1.58) | -0.74 (-3.06, 1.58) | 2.90 (0.14, 5.65) | 4.21 (0.12, 8.29) |
| ***Left ventricular systolic function*** |  |  |  |  |
| Left ventricular ejection fraction, % | -0.85 (-4.12, 2.41) | -0.85 (-4.12, 2.41) | -0.15 (-3.39, 3.09) | 0.12 (-5.51, 5.75) |
| Septal s’ velocity, cm/s | 0.0005 (-0.03, 0.04) | 0.0003 (-0.04, 0.04) | -0.04 (-0.08, 0.01) | -0.04 (-0.11, 0.03) |
| ***Left ventricular diastolic function*** |  |  |  |  |
| Transmitral E/e’ ratio | -0.08 (-0.67, 0.50) | -0.08 (-0.67, 0.50) | 0.27 (-0.37, 0.91) | -0.19 (-1.30, 0.92) |
| Septal e’ velocity, cm/s | -0.02 (-0.08, 0.05) | -0.02 (-0.08, 0.05) | -0.01 (-0.08, 0.06) | 0.06 (-0.06, 0.18) |
| Left atrium anteroposterior diameter index, mm/m | -0.02 (-0.09, 0.05) | -0.02 (-0.09, 0.05) | 0.05 (-0.04, 0.13) | 0.11 (-0.02, 0.24) |
| Transmitral E/A ratio | 0.03 (-0.07, 0.14) | 0.002 (-0.26, 0.26) | 0.21 (-0.04, 0.46) | 0.48 (-0.0009, 0.96) |
| Transmitral E deceleration time, ms | -2.34 (-12.04, 7.37) | -2.34 (-12.04, 7.37) | 8.76 (-3.83, 21.35) | -1.42 (-19.75, 16.91) |
| ***Right ventricular systolic function*** |  |  |  |  |
| Tricuspid annular plane systolic excursion, cm | 0.03 (-0.11, 0.16) | 0.03 (-0.11, 0.16) | 0.14 (-0.02, 0.30) | 0.24 (-0.01, 0.50) |
| ***Hemodynamic parameters*** |  |  |  |  |
| Stroke index, mL/m^2^ | -0.89 (-4.14, 2.35) | -0.89 (-4.14, 2.35) | 0.84 (-2.35, 4.03) | 1.65 (-3.70, 7.01) |
| Cardiac index, L/min⋅m^2^ | 0.01 (-0.09, 0.11) | 0.005 (-0.22, 0.23) | -0.20 (-0.43, 0.04) | -0.17 (-0.54, 0.21) |
| Central pulse pressure, mmHg | -1.39 (-4.12, 1.34) | -1.39 (-4.12, 1.34) | -1.12 (-4.60, 2.35) | 1.43 (-3.98, 6.84) |
| Mean arterial pressure, mmHg | 0.83 (-2.49, 4.16) | 0.76 (-2.29, 3.80) | -0.40 (-3.94, 3.15) | 6.14 (0.32, 11.95) |
| Heart rate, beats/min | -0.02 (-0.10, 0.07) | 0.83 (-2.49, 4.16) | -5.67 (-9.52,  -1.81) | -5.43 (-12.02, 1.15) |
| Total arterial compliance index, mL/mmHg⋅m^2^ | 0.76 (-2.29, 3.80) | -0.02 (-0.10, 0.07) | 0.02 (-0.25, 0.28) | 0.01 (-0.13, 0.16) |
| Systemic vascular resistance index, mmHg·min⋅m^2^/L | -0.06 (-4.05, 3.92) | -0.06 (-4.05, 3.92) | 2.78 (-1.57, 7.13) | 5.74 (-1.23, 12.70) |
| ***Vascular*** |  |  |  |  |
| Carotid intima-media thickness, mm | -0.004 (-0.02, 0.01) | 0.0007 (-0.02, 0.02) | 0.02 (0.005, 0.04) | 0.02 (-0.01, 0.05) |
| Carotid-femoral pulse wave velocity, m/s | -0.10 (-0.50, 0.29) | -0.10 (-0.50, 0.29) | 0.48 (0.02, 0.93) | 0.63 (-0.10, 1.36) |

Note: Values are reported as mean difference (95% confidence interval)

^a^Model 3 adjusted for age, sex, ethnicity, parents’ highest social class and young person’s education/employment/training status, body mass index, physical activity alcohol, smoking, mean arterial pressure, fasting lipids, glucose, insulin, C-reactive protein; mean arterial pressure was excluded in analyses of mean arterial pressure and systemic vascular resistance
